# Why are patients still in hospital after fast-track, unilateral unicompartmental knee arthroplasty

**DOI:** 10.1080/17453674.2020.1751952

**Published:** 2020-04-14

**Authors:** Christian Bredgaard Jensen, Anders Troelsen, Christian Skovgaard Nielsen, Niels Kristian Stahl Otte, Henrik Husted, Kirill Gromov

**Affiliations:** a Department of Orthopaedic Surgery, Copenhagen University Hospital Hvidovre;; b Clinical Orthopaedic Research Hvidovre, Copenhagen University Hospital Hvidovre, Denmark

## Abstract

Background and purpose — Previous studies have investigated risk factors related to prolonged length of stay following total knee arthroplasty (TKA), but little is known about specific factors resulting in continued hospitalization within the 1st postoperative days after unicompartmental knee arthroplasty (UKA). We investigated what specific factors prevent patients from being discharged on the day of surgery (DOS) and the first postoperative day (POD-1) following primary UKA in a fast-track setting.

Patients and methods — We prospectively collected data on 100 consecutive and unselected medial UKA patients operated from December 2017 to May 2019. All patients were operated in a standardized fast-track setup with functional discharge criteria continuously evaluated from DOS and until discharge.

Results — Median length of stay for the entire cohort was 1 day. 22% and 78% of all patients were discharged on DOS and POD-1, respectively. Lack of mobilization and pain separately delayed discharge in respectively 78% and 24% of patients on DOS. The main reasons for lack of mobilization were motor blockade (37%) and logistical factors (26%). For patients placed 1st or 2nd on the operating list, we estimate that the same-day discharge rate would increase to 55% and 40% respectively, assuming that pain and mobilization were successfully managed.

Interpretation — One-fifth of unselected UKA patients operated in a standardized fast-track setup were discharged on DOS. Pain and lack of mobilization were the major reasons for continued hospitalization within the initial postoperative 24–48 hours. Strategies aimed at decreasing length of stay after UKA should strive to improve analgesia and postoperative mobilization.

The number of unicompartmental knee arthroplasties (UKAs) performed in patients suffering from osteoarthritis has steadily increased. UKA has the potential benefit of not only improving patient-reported outcomes, but also to reduce morbidity, complications, and cost (Liddle et al. 2014, Beard et al. 2019). In the United Kingdom, 9% of all primary knee arthroplasties performed in 2018 were UKAs while this number is as high as 20% in Denmark (Danish Knee Arthroplasty Register 2019, National Joint Registry for England 2019).

UKA is effective and safe when performed in a fast-track setting and outpatient UKA in selected patients has been shown to be feasible and safe (Munk et al. 2012, Cross and Berger 2014, Bovonratwet et al. 2017, Kort et al. 2017). However, the number of patients actually being discharged on DOS that were scheduled for outpatient surgery differs between studies and ranges from 37% to 100% (Gondusky et al. 2014, Bradley et al. 2017, Jenkins et al. 2019, Rytter et al. 2019).

Studies have shown an association between increased length of stay (LOS) and an increase in both complication and readmission rates (Otero et al. 2016). In order to reduce LOS and increase patient satisfaction, a focus on successfully managing well-defined discharge criteria in a multimodal approach is imperative (Husted et al. 2008, Cross and Berger 2014). In addition, decreased LOS and outpatient procedures are associated with financial benefits, which have further fueled interest in decreasing LOS and ensuring DOS discharged following UKA (Bradley et al. 2017). Finally, decreased LOS is also shown to increase patient satisfaction levels (Reilly et al. 2005, Richter and Diduch 2017).

A study has been conducted to explore reasons for prolonged hospitalization in a fast-track setting following TKA (Husted et al. 2011). However, in spite of a growing number of UKAs performed each year, no study explicitly exploring reasons for prolonged hospitalization beyond DOS following UKA in a fast-track setting has been published at present.

Therefore, we investigated reasons for continued hospitalization beyond DOS following UKA in a fast-track setting.

## Patients and methods

100 consecutive and unselected patients undergoing primary, unilateral, medial UKA in one institution between December 2017 and May 2019 were enrolled in this study. All patients were operated with cementless mobile bearing implants using microplasty instruments with a minimal invasive technique as described by the manufacturer. Patients were operated on by 4 surgeons, all using UKA in above 20% of all performed knee arthroplasties. Tourniquet was used during the entire surgery, set at 100 mmHg above the individual systolic pressure. Patients were intended to be operated using spinal anesthesia (SA) with 2 mL 0.5% hyperbaric bupivacaine, unless the patient specifically requested general anesthesia (GA) or GA was chosen by the anesthesiologist due to patient characteristics. If GA was chosen, remifentanil and propofol was used.

All patients received local infiltration analgesia (LIA) with 200 mL 0.2% ropivacaine injected in the posterior capsule, periarticular tissues, and subcutaneous tissue. Standard 3-layer closure with tissue adhesive was performed and a compression bandage was applied to the limb (Andersen et al. 2008, Gromov et al. 2019). All patients received a single-shot intravenous injection of 125 mg methylprednisolone 30 minutes before the beginning of the surgery together with 2 g dicloxacillin.

Pain medication included paracetamol 1 g and celecoxib 200 mg as single doses preoperatively, and paracetamol 1g x 4 and celecoxib 200 mg x 2 daily for 7 days postoperatively. No opioids were given routinely, and morphine 5 mg was given as rescue medication only.

Upon completed surgery, patients operated with SA with ASA 1 and 2 were transferred directly to the patient ward, while patients with ASA > 2 and patients operated using GA were transferred to the post-anesthesia care unit (PACU), where they stayed until fulfilling modified Aldrete discharge criteria (Aasvang et al. 2017). Mobilization was attempted upon patients’ return to the ward as soon as motor function allowed. Physiotherapy was focused on reaching functional discharge criteria without any requirements for specific range of motion prior to discharge. Postoperative radiographs were taken on the DOS or the day after, if the patient was not discharged on DOS.

All patients were evaluated continuously with regard to functional discharge criteria. For patients who were not discharged, the reason for not fulfilling the discharge criteria was recorded at 8 pm on the day of surgery, and at 2 pm on postoperative day 1 (POD-1) and postoperative day 2 (POD-2). Fulfillment of the following criteria was recorded and included: independent mobilization, postoperative nausea and vomiting (PONV), circulatory insufficiency, pain, wound issues, and urinary retention. If patients were not sufficiently mobilized, the following possible underlying reasons were recorded and registered as 1 or more of following: logistics, motor blockade, PONV, pain, muscle weakness, or dizziness. Logistics included lack of postoperative radiographs and limited access to physiotherapy due to organizational factors. Acceptable pain levels were < 5 VAS at rest and < 7 VAS during physical activity. Patients were regarded as circulatory stabile with a pulse < 90 and systolic blood pressure > 100. Wound issues covered bleeding from the surgical wound. Urinary retention was evaluated using a bladder scanner, with 800 mL being the cutoff for catheterization.

### Ethics, funding, and potential conflicts of interest

Approval from the ethical committee was not required since this was purely an observational study. Data access was approved by the national data committee (HVH-2012-048). This research did not receive any financial support. The authors declare no conflicts of interest.

## Results

Patient demographics are given in Table 1.

**Table 1.** Patient demographics

Factor	n	mean (range)
Sex	Female	57
	Male	43
	Total	100
BMI		30 (21–53)
Age		67 (39–93)
ASA score	1–2	80
	3–4	20
Anaesthesia	General	11
	Spinal	89

Among all patients, 22% were discharged on DOS, with 78% discharged on POD-1, and 98% on POD 2. Of the 22 patients (22%) discharged on DOS, 18 patients were ASA 1–2, and the remaining 4 patients were ASA 3–4.

Of the 11 patients operated using GA, 3 were discharged on DOS, 5 on POD-1, and 3 on POD-2.

When only looking at patients operated as #1 or #2 on the surgery schedule, 27% of patients were discharged on DOS and 80% and 99% of patients discharged on POD-1 and POD-2, respectively. Median LOS for the entire group was 1 day (range 0–3).

Lack of mobilization (81%), pain (19%), and urinary retention (18%) were the main reasons for patients not meeting the discharge criteria on DOS (Table 2). When only looking at patients operated as #1 and #2, urinary retention was an issue in 11% of patients, with lack of mobilization (78%) and pain (24%) being the most important reasons for not being discharged. PONV, circulatory insufficiency, and wound issues were present in 1–6% of all patients (Table 2).

**Table 2.** Reasons for not being discharged displayed as count and percentage of total amount of patients still hospitalized on day of surgery (DOS), postopertive day 1 (POD-1) and postopertive day 2 (POD-2)

	Not discharged on		
	DOS	POD-1	POD-2
Reasons	n = 78	n = 22	n = 2
Lack of mobilization	63 (81)	15 (68)	1 (50
PONV	5 (6)	2 (9)	2 (100)
Circulatory insufficiency	1 (1)	0 (0)	0 (0)
Pain	15 (19)	11 (50)	0 (0)
Wound issues	2 (3)	4 (18)	1 (50)
Urinary retention	14 (18)	1 (5)	0 (0)

Several reasons for patients not being sufficiently mobilized were recorded. In patients with mobilization issues still hospitalized on DOS the major reasons were motor blockade (44%), logistics (24%), and pain (19%) (Table 3). 1 patient operated using GA was registered as insufficiently mobilized due to motor blockade.

**Table 3.** Reasons for lack of mobilization displayed as count and percentage of total amount of patients lacking mobilization on day of surgery (DOS), postopertive day 1 (POD-1) and postopertive day 2 (POD-2)

	Not mobilized on		
	DOS	POD-1	POD-2
Reasons	n = 63	n = 15	n = 2
Logistics	15 (24)	1 (7)	0 (0)
Motor blockade	28 (44)	0 (0)	0 (0)
PONV	4 (6)	0 (0)	1 (50)
Pain	12 (19)	9 (60)	0 (0)
Muscle weakness	3 (5)	2 (13)	0 (0)
Dizziness	1 (2)	3 (20)	0 (0)

When looking only at patients operated as #1 or #2 on the surgery schedule, the percentage of patients not mobilized sufficiently due to motor blockade decreases to 37%, while the percentage of patients not mobilized sufficiently due to pain increases to 26%. PONV, muscle weakness, and dizziness were infrequent reasons for lack of mobilization.

Assuming the lack of mobilization could be managed successfully in patients operated as #1 or #2 on the surgery schedule, we estimate an increase in discharge percentage up to 55% on DOS. Sorted for the specific issues resulting in lack of mobilization, an additional 11 and 9 patients could possibly be discharged, when assuming successful management of motor blockade and logistics, respectively, and thus potentially increasing DOS discharge to 41% and 39%, respectively. Assuming pain could be managed successfully in patients operated as #1 or #2 on the surgery schedule, we estimate an increase in discharge percentage up to 40% on DOS (Table 4).

**Table 4.** Possible improved discharge percentage on the day of surgery if the individual discharge criteria was managed successfully. Not DOS discharge are the number of patients failing to meet only that specific discharge criteria

	Not DOS discharge ^a^ n	Possible DOS discharge ^a^**^, b^** n (%)
Actual DOS discharge ^a^	–	20 (27)
Cause of not DOS discharge		
Lack of mobilization ^c^	21	41 (55)
PONV	4	24 (32)
Circulatory insufficiency	0	20 (27)
Pain	10	30 (40)
Wound issues	0	20 (27)
Urinary retention	1	21 (28)

**^a^**1st and 2nd patient on program.

**^b^**Possible DOS discharge is the sum of Actual DOS discharge and

Not DOS discharge

**^c^**Patients not mobilized due to pain or PONV were excluded from

lack of mobilization and included in pain and PONV, respectively.

## Discussion

In this prospective single-center study, we investigated specific factors responsible for continued hospitalization following medial UKA in a fast-track setting. Reasons for not being discharged on the DOS were primarily lack of mobilization and pain. Primary reasons for lack of mobilization were motor-blockade, pain and logistics.

We found that 22% of unselected patients were discharged on DOS. A study by Jenkins et al. (2019) reported 39% (n = 669) of consecutive unselected unilateral UKA patients to be discharged on DOS. The aim of that study was to investigate the effect of delaying knee flexion on different outcome measures such as LOS. Some important changes in the postoperative protocol in the study were delaying knee flexion and a physiotherapist working late shifts. Also, efforts were made to have UKA patients scheduled for surgery as #1 if possible. Jenkins et al. (2019) reported reduced muscle strength to be the most common reason for continued hospitalization as well as dizziness and nausea. A study by Bradley et al. (2017) reported a day of surgery discharge percentage of 85% (n = 72). Patients in that study were included in the DOS discharge group only if they were cleared by a preoperative team after testing and optimizing their coexisting medical conditions. Another study found 85% of patients (n = 20) to be discharged on DOS following UKA, when including patients only if they had no severe cardiologic, pulmonary, internal disease, or fear of an outpatient procedure (Kort et al. 2017). The reason for these exclusion criteria was that such patients might need postoperative adjustment of medication resulting in a delayed discharge. Excluded patients were operated in a rapid-recovery inpatient pathway. A study including 160 outpatient UKA patients reports 100% of patients to be discharged on DOS (Gondusky et al. 2014). All patients had to be cleared by their primary care physician; however, the exact medical reasons for exclusion are not fully described. Following discharge, patients were seen by a physiotherapist at home, starting from POD-1 and then 3 times a week for 3 weeks.

We found a substantially lower number of UKA patients discharged on DOS compared with the studies mentioned above. In the case of Jenkins et al. (2019) the main reason for this difference might be that we have introduced no changes to our postoperative protocol in relation to this study, thus not increasing the focus on LOS as measurement of performance within the department. Also, no changes were made regarding available resources such as a physiotherapist working late or UKA patients being scheduled for early surgery slots. The main reason for the difference between our study and the other studies cited above is most likely patient selection, as these other studies apply specific inclusion criteria for outpatient patients, while we investigated all consecutive and unselected patients deemed eligible for UKA. This resulted in a higher mean ASA score in patients compared with the above-mentioned studies, and a very large range in both patient age and BMI. This large range in patient demographics in an unselected cohort was also reported by Jenkins et al. (2019). Furthermore, all patients in our study were discharged to their own homes with no additional care. The difference in the amount of postoperative care needed in unselected and selected patient groups could explain the difference in length of stay. While allocating patients to outpatient and inpatient settings would potentially allow for an optimized postoperative approach focused on patients with a high possibility of discharge on DOS, this was not the aim of our study. We included all patients with the purpose of identifying possible factors preventing early discharge regardless of specific patient characteristics.

Pain was an issue in 24% of cases in our study, which is lower compared with a similar study investigating TKA conducted in the same surgical department in 2011, which found pain to be an issue in 53% of cases (Husted et al. 2011). This finding is expected as UKA is a less invasive procedure compared with TKA, resulting in less immediate postoperative pain.

The multimodal opioid-sparing analgesic regime in our setting consists of a single-shot intravenous injection of 125 mg methylprednisolone as well as paracetamol 1g x 4 and celecoxib 200 mg x 2 daily for 7 days postoperatively with morphine used as rescue medication only.

A preoperative injection of methylprednisolone has been shown to reduce pain at rest, pain during walking, and opioid consumption in the first 24 hours after UKA surgery (Rytter et al. 2017). Similar results, including a reduction in PONV and ondansetron use, have been shown in the first postoperative 48 hours following TKA surgery (Lunn et al. 2011). The use of methylprednisolone preoperatively is also part of a strategy to reduce PONV, which was present in only 6% of our patients still hospitalized on DOS. The use of midodrine has been suggested to decrease orthostatic hypotension and subsequently nausea and dizziness but was found to have limited effect (Jans et al. 2015).

LIA is a part of our intraoperative procedure because it has been found to reduce postoperative pain following TKA surgery, and also when combined with a multimodal opioid-sparing analgesic regime (Andersen and Kehlet 2014, Seangleulur et al. 2016). In regard to UKA, a study found LIA to improve postoperative pain management and reduce opioid consumption and LOS (Essving et al. 2009). That study, however, did use a different composition of drugs compared with our LIA regime.

Improved pain management could possibly be achieved using opioids or peripheral nerve blocks as part of the standard analgesic regime, but adverse effects such as an increased risk of falls upon initiation of opioid use as well as sedation, delirium, nausea, and urinary retention have been reported (Golladay et al. 2017, Seppala et al. 2018). Also, peripheral nerve blocks have limited additional value alongside LIA (Gudmundsdottir and Franklin 2017). Our postoperative strategy following fast-track knee surgery focuses on achieving sufficient and early mobilization, which could be impaired due to adverse effects of increased opioid consumption. We are therefore inclined to accept a higher level of pain in exchange for better postoperative mobilization.

Reduction of pain is crucial, but it is not only desirable as a short-term goal regarding discharge as a recent study found control of early postoperative pain to be associated with improved 2-year functional outcome following UKA (Lakra et al. 2019).

Among patients not discharged due to insufficient mobilization on DOS, a motor blockade was registered as the main reason in 44% of patients. Management of motor blockade in patients operated as #1 or #2 would allow for additional discharge of 11 patients on DOS. Interestingly, 1 patient operated using GA was reported to have motor blockade suggesting difficulty distinguishing between muscle weakness and “true” motor blockade. Therefore, the proportions of motor blockade and muscle weakness reported in our study might also be a product of difficulty distinguishing between the two. It is possible to speculate that GA might be better suited for UKA surgery with intended DOS discharge, though there is insufficient evidence to advocate for one over the other (Kehlet and Joshi 2019).

Since logistical factors such as limited access to physiotherapy impacted 24% of insufficiently mobilized patients on DOS, organizational factors are to be further optimized. Physiotherapy is an important factor in short-term recovery since a loss of quadriceps function close to 80% is reported after knee arthroplasty (Bandholm and Kehlet 2012). Yet, due to limited literature no specific best practice has currently been determined. Even so, one aspect of acute postoperative rehabilitation has been determined: early initiation of physiotherapy after both UKA and TKA is associated with a reduction in LOS and overall cost, with no increase in adverse effects (Masaracchio et al. 2017, Henderson et al. 2018). However, the need for intense and early physiotherapy could be the reason why it is an organizational challenge. As previously mentioned, Jenkins et al. (2019) reported a potential reduction in LOS after UKA, due to a delay in knee flexion and physiotherapists working late shifts. However, that study does not include a control group.

Our study has several limitations. Mainly, our results might have limited external validity since alternative setups regarding anesthesia, perioperative care, and treatment could impact reasons for continued hospitalization. However, our fast-track setup is well described and our study investigates an unselected patient population, increasing the ability for other surgical centers to compare their results with ours. Also, all patients eligible for UKA surgery were considered eligible for DOS discharge. Additionally, both GA and SA were used, but no specific distinctions between the 2 groups were made, even though differences between the groups might be present.

## Conclusion

One fifth of unselected UKA patients operated in a standardized fast-track setup were discharged on DOS. Pain and lack of mobilization were the major reasons for continued hospitalization within the initial postoperative 24–48 hours. To improve the number of patients discharged on the day of surgery, initiatives should focus on improving postoperative mobilization and postoperative pain management.
